# Histological Evaluation of Chemo Mechanical Caries Removal with a Babaco-Based Formulation Gel

**DOI:** 10.3390/gels11040257

**Published:** 2025-04-01

**Authors:** María del Carmen Pariona-Minaya, Melissa Berrezueta-Pérez, Gerson Cabezas-Bernhardt, Ebingen Villavicencio-Caparo

**Affiliations:** 1 Faculty of Dentistry, University Peruana Cayetano Heredia, Lima 15102, Peru; mparionam@ucacue.edu.ec; 2 Health and Wellness Department, Faculty of Dentistry, Catholic University of Cuenca, Cuenca 010107, Ecuador; melissaelizabethbp@gmail.com; 3 PhD. Program Alumnni, Faculty of Dentistry, University Peruana Cayetano Heredia, Lima 15102, Peru; gcabezas@usfq.edu.ec; 4 Health Science Department, Faculty of Dentistry, University San Francisco de Quito, Quito 170901, Ecuador; 5 Research Group, Health and Wellness Department, Faculty of Dentistry, Catholic University of Cuenca, Cuenca 010107, Ecuador

**Keywords:** papain, dental caries, histology

## Abstract

The study investigated the effectiveness of a babaco-based gel derived from an endemic Ecuadorian fruit for chemomechanical caries removal compared to the conventional non-rotary mechanical method. Babaco contains proteolytic enzymes that soften decayed dental tissue, making it a potential alternative for caries treatment. An ex vivo experimental study was carried out using sixty extracted human teeth, each divided into two sections: one treated with babaco gel and the other with a spoon excavator. Four extraction methods (maceration, distillation, Soxhlet, and percolation) were used to prepare the gel. After rehydration and submersion in nitric acid, an oral pathologist evaluated the efficacy of caries removal. Results showed a 78% success rate for the babaco-treated sections, significantly higher than the 14% success rate for the conventional method. The distillation-extracted gel subgroup performed best at *p* < 0.01 Fisher Chi^2^. The study concluded that babaco gel is more effective for caries removal than traditional methods, highlighting its potential as a chemical-mechanical alternative.

## 1. Introduction

Dental caries, a multifactorial and highly prevalent disease affecting individuals of all ages, remains one of the most commonly treated conditions in dental practice. The removal of carious tissue is a fundamental procedure, with treatment options ranging from conventional methods to minimally invasive techniques [1]. Conventional approaches typically involve the use of high-speed rotary instruments equipped with sharp dental burs. However, these methods carry significant drawbacks, such as the risk of overheating the tooth during caries removal, which can potentially damage the dental pulp [2]. Additionally, conventional techniques often result in the over-preparation of cavities due to the unnecessary removal of healthy dentin, compromising the structural integrity of the tooth [2,3]. These limitations have spurred the development and adoption of alternative, less invasive approaches to caries management.

Atraumatic restorative treatment (ART) is one such alternative, involving the manual removal of caries using dentin spoons or excavators. This method is less invasive and more selective than traditional techniques, relying on tactile perception to preserve healthy tissue [4]. Although ART requires more clinical time, it significantly reduces patient anxiety and fear, making it a favorable option for certain populations [2]. To enhance ART, chemical agents that selectively act on carious dentin have been introduced. These chemomechanical caries removal (CMCR) techniques begin with the softening of infected dentin, preserving affected dentin that has the potential for remineralization. Commonly used chemical agents include sodium hypochlorite and proteolytic enzymes such as papain [2,5].

Papain, a proteolytic enzyme derived from papaya, is the most widely used enzyme in CMCR. It hydrolyzes type I collagen in dentin, facilitating the removal of carious tissue [6]. Since 2003, papain-based gels like Carisolv^®^, Papacarie^®^, and Brix3000^®^ have been commercially available [7,8]. Papain is also found in other fruits, such as figs, pineapple, kiwi, and babaco (*Carica pentagona*), a fruit endemic to Ecuador and closely related to papaya [6,9,10]. Babaco, in particular, has shown promise due to its similar enzymatic properties, making it a potential source for developing CMCR gels [9,11,12].

Recent research has focused on optimizing caries removal techniques to preserve as much healthy dentin as possible. A 2015 systematic review by Hamama et al. compared the time required for caries removal using different methods. They found that rotary drills averaged 2.99 min, while Papacarie^®^ and Carisolv^®^ required 6.98 and 8 min, respectively [13]. Despite the longer procedural time, CMCR methods like Carisolv^®^ were associated with reduced pain levels and a decreased need for anesthesia, as demonstrated by Maru et al. in the same year [14,15]. These findings have been consistently supported by studies conducted globally over the past decade, reinforcing the reliability of CMCR as a viable alternative to traditional methods [16].

The effectiveness of caries removal and the success of subsequent restorative procedures remain critical considerations in dental research. Studies comparing CMCR to conventional methods have yielded mixed results. For instance, Lie et al. in 2014 found no statistically significant difference in caries removal rates between Carisolv^®^ and rotary instruments, suggesting comparable efficacy [16]. Similarly, da Silva et al. demonstrated that combining ART with CMCR was effective and led to higher patient satisfaction, though ART alone did not significantly reduce pain or discomfort [17]. In contrast, Senthilkumar and Ramesh in 2020 and Ferreira et al. in 2023 concluded that conventional rotary burs remain more reliable than alternative methods, emphasizing the need for further rigorous clinical trials to validate emerging techniques [18,19].

Emerging technologies, such as Smart burs, have also been explored. Mehrotra et al. found that Smart burs exhibited superior microbiological efficacy and efficiency, positioning them as a potential alternative to CMCR systems for treating primary molars. However, it is cautioned that further studies are needed to confirm their long-term effectiveness [20]. Additionally, CMCR agents have demonstrated notable antimicrobial effects against cariogenic bacteria, as highlighted by Janvier et al. [20]. Regarding restoration failure, Rossoni et al. found no significant difference between chemomechanical and mechanical excavation methods [21]. Schwendicke et al. further noted that avoiding the complete removal of softened or stained dentin may reduce the risk of complications, underscoring the clinical benefits of less invasive techniques [22].

This study aimed to isolate the active ingredient from babaco (*Carica pentagona*) and formulate a gel for dental applications, ensuring optimal viscosity for cavity adherence and a color contrast with dentin for easy identification. The babaco-based gel offers novelty in chemomechanical caries removal (CMCR) due to its unique enzymatic profile, local availability, and optimized extraction methods. Babaco latex contains high levels of proteolytic enzymes, including papain, with activity peaking at 8125.65 U/g in 5-month-old fruits, making it a potent alternative to papain-based gels like Carisolv^®^ and Papacarie^®^. Additionally, babaco’s enzymatic spectrum includes lipolytic enzymes, broadening its potential applications [23].

Babaco’s diverse enzymatic activity, particularly α-mannosidase and proteases, enhances its effectiveness in breaking down carious tissue compared to single-enzyme agents. Its dual mechanism targets both polysaccharide and protein components, potentially improving caries removal efficiency. As an endemic fruit of Ecuador, babaco provides a sustainable, locally sourced alternative to imported enzymatic agents, supporting regional economies [24]. Furthermore, babaco may offer antifungal benefits, similar to its relative papaya, potentially aiding in combating oral fungal pathogens like Candida albicans [25,26].

The babaco-based gel’s development presents clinical advantages, including enhanced enzymatic activity, potential antifungal properties, and cost-effective production. Its dual-action capability could streamline treatments and improve outcomes, particularly in high-risk populations. This study pioneers the evaluation of babaco-based gel efficacy against conventional hand excavation. The proteolytic enzymes were extracted using methods such as Soxhlet extraction, maceration, distillation, and percolation. These techniques were chosen for their well-documented efficacy in preserving enzymatic activity, accessibility, and cost-effectiveness, ensuring practical application within the study’s context [5,10,27,28].

By comparing the babaco-based gel to traditional methods, this research aims to advance minimally invasive dental care, offering a sustainable and effective alternative for caries management, particularly in regions where babaco is cultivated [5,10,27,28].

## 2. Results and Discussion

### 2.1. Results

The sample consisted of 15 histological sections made using the maceration method, 15 using the distillation method, 15 using the Soxhlet method, and 13 using the percolation method. In the latter method, two samples were lost when the histological section was made, thereby reducing the *n* to thirteen for percolation.

**Figure 1:**
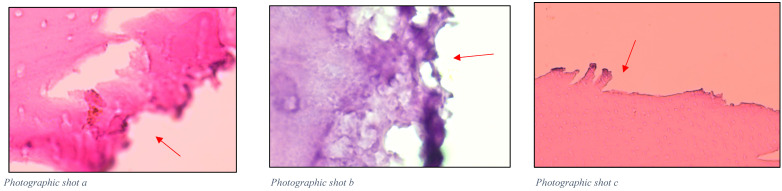
Histological sections of teeth treated with conventional dentin spoon excavation. Photographs (**a**–**c**) show the left side of the teeth, highlighting irregular tissue tearing and residual infected dentin after treatment with the conventional method. The areas with the most tearing are highlighted by the arrows.

**Figure 2:**
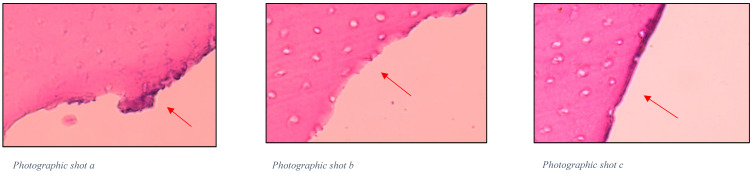
Histological sections of teeth treated with babaco-based gel. Photographs (**a**–**c**) show the right side of the teeth, demonstrating more regular tissue removal and preservation of healthy dentin after treatment with the babaco gel. The arrows indicate areas with less tearing compared to Figure 1.

Histologically, in Figure 1, irregular tissue tearing can be observed in the areas where the conventional method was applied. Additionally, a portion of infected dentin remains present. The sections treated with the papain gel appear more regular, and the remaining dentin is mostly healthy tissue, as observed in Figure 2.

The microscopic analysis of the teeth showed that the side that received the Babaco gel had an overall success rate of 78%, and the side without the active ingredient had a success rate of 14% in removing carious tissue, which means there was a statistically significant difference.

**Figure 3:**
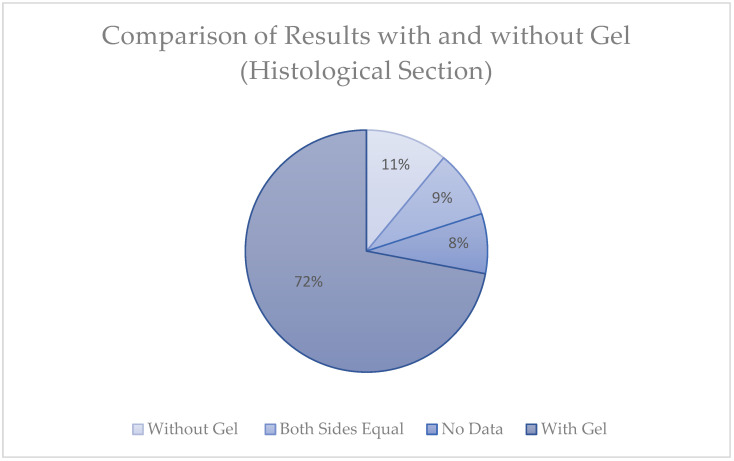
Distribution of histological outcomes comparing treatment with and without gel. A significant proportion (72%) of cases showed improvement with gel, whereas 8% did not improve (without gel), 9% lacked data, and 11% showed no difference between sides.

A comparative analysis was also conducted to evaluate the efficacy of gel application in histological sections, as illustrated in Figure 3. The results indicate that 72% of the samples exhibited superior outcomes on the side treated with the gel, while 11% showed better results on the untreated side. Among the remaining cases, 8% demonstrated equivalent results between the gel-treated and untreated sides, and 9% of the data were non-evaluable.

#### 2.1.1. Extraction Methods

Regarding comparing the gel extraction methods, a 100% success rate in caries removal was found with the distillation method, followed by 85% with the percolation method. The sides without gel obtained 0% and 23%, respectively (Table 1).

**Figure 4:**
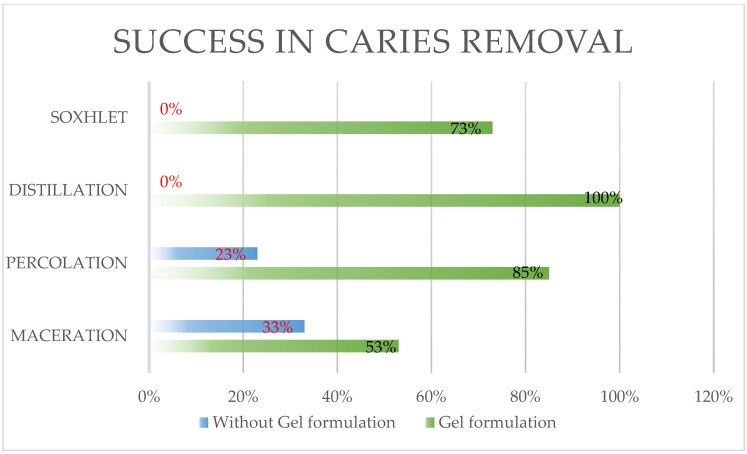
Success rates of caries removal based on extraction methods. A bar chart comparing the success rates of caries removal for the babaco gel across four extraction methods: distillation (100%), percolation (85%), Soxhlet (73%), and maceration (53%).

For the Soxhlet group, 73% of the samples with active ingredients succeeded, with total failure on the side without gel (Figure 4).

Finally, in the maceration group, a 53% success rate was found on the side with gel, while the side with the conventional method obtained 33% success in caries removal (Figure 4).

#### 2.1.2. Statistical Analysis

To test the hypothesis, Fisher’s chi-square test was used with 95% reliability α < 0.05 in the case of tables with less than five units per box and Pearson’s chi-square test was used in the table with boxes exceeding five study units.

Table 2 presents the results of a Post Hoc test, comparing the success and failure rates of caries removal across the four different extraction methods used: distillation, maceration, percolation, and Soxhlet. The distillation method demonstrated the highest success rate, with all 15 samples (100%) successfully removing caries, placing it in Post hoc Group A. The percolation and Soxhlet methods also showed relatively high success rates, with 11 out of 13 (85%) and 11 out of 15 (73%) successful cases, respectively, both classified in Post hoc Group B due to their similar values. In contrast, the maceration method had the lowest success rate, with only 8 out of 15 (53%) successful cases, placing it in Post hoc Group C. The Chi-square test yielded a *p*-value of 0.019, indicating a statistically significant difference in success rates among the extraction methods. The residuals further highlight the deviations from expected values, with distillation showing the most favorable outcomes and maceration the least. These results suggest that the distillation method is the most effective for extracting the active components of the babaco gel, while maceration is the least effective, likely due to differences in enzymatic preservation and extraction efficiency.

### 2.2. Discussion

The findings of this study, which demonstrate a 78% success rate in caries removal using a babaco-based gel, are consistent with several previous studies that have evaluated the efficacy of chemomechanical caries removal (CMCR) techniques. However, there are also notable differences in outcomes compared to other research, particularly regarding the extraction methods and the comparative efficacy of CMCR versus conventional non-rotary methods.

#### 2.2.1. Efficacy of Chemomechanical Caries Removal

The overall success rate of 78% for the babaco-based gel in this study aligns with the findings of Saber et al. in 2020, who reported that CMCR techniques, particularly those using papain-based gels, are more effective at the histological level than conventional curettage [29,30,31]. Similarly, a study by Santos et al. that same year found that CMCR methods, including those using Carisolv^®^ and Papacarie^®^, effectively removed carious tissue while preserving healthy dentin, with success rates ranging from 70% to 85% [1,2]. A prospective study by Bussadori with a 14-month follow-up after using the Papacarie^®^ caries removal agent demonstrated success in 13 out of 14 cases. This study was conducted on permanent teeth, with both clinical and radiographic follow-up. The findings highlight the relevance of this minimally invasive technique and its potential for further development, creating a favorable scenario for the future industrial development of Babaco gel in Ecuador. These results suggest that the babaco-based gel performs comparably to commercially available CMCR products [31].

In 2022, Bratu et al. published a bibliometric analysis spanning 10 years of publications, identifying 58 studies that utilized CarisolvTM and BRIX 3000^®^. Their research emphasized the efficacy of methods such as BRIX3000^®^ and CarisolvTM in removing infected dentin in both deciduous and permanent teeth, demonstrating that these methods are well-tolerated by patients. Given that these are two distinct commercial preparations, it can be inferred that any other preparation with a similar mechanism of action would yield comparable results in terms of patient tolerance. Bratu et al. also evaluated the efficacy of various caries removal techniques in terms of pain and anxiety during treatment, concluding that BRIX3000^®^ and CarisolvTM were well-tolerated methods [6].

Another important observation by Bratu et al. is that Papacárie^®^ and CarisolvTM stimulate human pulp fibroblasts to produce extracellular matrix proteins. This suggests a positive impact on the efficacy of these methods, as it refers to the process by which these specialized cells in pulp tissue synthesize and secrete proteins that form part of the surrounding extracellular matrix. This creates a three-dimensional network of proteins and carbohydrates that provides structural support and regulates various cellular functions. This indicates the potential of these preparations to interact favorably with pulp tissue cells, which may have implications for the biological response of pulp tissue and their ability to promote dental tissue regeneration and repair [6].

However, the present study’s results contrast with those of Cardoso et al., who found that in previous studies, just using spoon excavators was more effective than CMCR in some cases, particularly for bacterial reduction [32]. This discrepancy may be due to differences in the gels’ formulation or the specific extraction methods employed. For example, the babaco-based gel in this study achieved a 100% success rate when the active ingredient was extracted via distillation. This may explain its superior performance compared to some CMCR products.

#### 2.2.2. Extraction Methods and Their Impact on Efficacy

The study compared four extraction methods—maceration, distillation, Soxhlet, and percolation—and found that the distillation method yielded the highest efficacy (100% success rate), followed by percolation (85%), Soxhlet (73%), and maceration (53%). These results are consistent with the findings of Moya et al., who reported that the distillation method was more effective in preserving proteolytic agents’ enzymatic activity than other extraction techniques [33]. Similarly, a study by Guedes et al. found that the extraction method significantly influenced the efficacy of CMCR agents, with distillation and percolation methods showing the highest success rates [27].

In contrast, in their study, Romero et al. did not find significant differences between the distillation method and conventional curettage [34]. This may be due to variations in the gel’s formulation or the specific conditions under which the extraction was performed [34]. This highlights the importance of optimizing extraction methods to maximize the efficacy of CMCR agents.

#### 2.2.3. Comparative Efficacy with Conventional Curettage

The present study found that the conventional method had a success rate of only 14%, significantly lower than the 78% success rate achieved with the babaco-based gel. This finding is consistent with several studies that have compared CMCR techniques to conventional methods. For example, a systematic review by Souza et al. in 2022 concluded that CMCR methods, including those using papain-based gels, are generally more effective than conventional ones in preserving healthy dentin and reducing patient anxiety [34].

However, some studies have reported mixed results. Sharma et al. found no significant difference in caries removal efficacy between CMCR and conventional non-rotary excavation methods, although they noted that CMCR was associated with lower patient anxiety levels [15,30]. Similarly, Bogetta et al. reported that the effectiveness of the conventional method was comparable to that of CMCR, suggesting that the choice of method may depend on the specific clinical context [35].

Regarding clinical research, Bratu et al. studied the adhesion of composite resin to caries-affected dentin after treatment with CarisolvTM, providing insights into the efficacy of this method in preparing for restorations. These findings support the efficacy and feasibility of chemo-mechanical caries removal methods using BRIX3000^®^ and CarisolvTM in various clinical and research contexts. This is particularly important, as it is already known that chemo-mechanical caries removal using enzymes does not compromise the adhesion of dentin adhesives used in dental restorations [6].

#### 2.2.4. Patient-Centered Outcomes

One of the key advantages of CMCR, as demonstrated in this study, is its potential to reduce patient anxiety and discomfort. The babaco-based gel was applied for only 60 s, and the procedure was less invasive than conventional curettage, which aligns with the findings of Fronza et al. and Ghanem et al., reporting that CMCR techniques are particularly beneficial for pediatric patients due to their minimally invasive nature [36,37]. Similarly, a study by Sierra et al. (2019) found that using CMCR products like Brix3000^®^ significantly reduced anxiety and fear in pediatric patients compared to traditional rotary methods [38].

#### 2.2.5. Limitations and Future Directions

This study represents the first approach to developing a babaco-based gel for applications such as chemomechanical caries removal. While the results are promising, particularly in terms of the high proteolytic activity observed in babaco latex, the study is primarily focused on establishing the enzymatic profile and initial efficacy of the gel. As such, the research provides preliminary results that highlight the potential of babaco latex as a source of proteolytic enzymes but does not address several critical aspects necessary for its clinical and commercial viability.

One significant limitation is the lack of investigation into the gel’s long-term stability and storage conditions. Factors such as temperature, humidity, and exposure to light could significantly impact the stability of the enzymes in the gel. For example, proteolytic enzymes like papain, which are structurally similar to those in babaco latex, are known to degrade over time, especially under suboptimal storage conditions. Therefore, future studies must evaluate the gel’s stability under various storage conditions to determine its shelf life and optimal storage parameters. Rigorous testing, including accelerated aging studies, will be essential to simulate long-term storage and predict the gel’s performance over time.

Additionally, the study was conducted ex vivo, and the efficacy of the babaco-based gel in a clinical setting remains to be determined. Clinical trials are needed to evaluate the gel’s performance in vivo, particularly in pediatric patients who may benefit most from minimally invasive caries removal techniques. Furthermore, the study focused on the efficacy of caries removal but did not evaluate long-term outcomes, such as the remineralization potential of the treated dentin or the bond strength of restorative materials applied after CMCR. Future studies should explore these aspects to provide a more comprehensive understanding of the babaco-based gel’s potential.

Another important consideration is the antimicrobial properties of the babaco-based gel. Given the role of bacteria in the progression of dental caries, it would be valuable to assess whether the gel has any antimicrobial effects that could further enhance its efficacy. Addressing these limitations in future research will be crucial for establishing the gel’s clinical and market viability, ensuring it retains its enzymatic efficacy throughout its shelf life, and providing a more comprehensive understanding of its potential benefits and applications.

#### 2.2.6. Clinical Applicability

The babaco-based gel represents a significant advancement in the field of chemomechanical caries removal, offering benefits for both pediatric and adult patients. Its minimally invasive nature, ability to reduce anxiety and pain, and potential cost-effectiveness make it a valuable tool for modern dental practices. For pediatric patients, the gel provides a comfortable and effective alternative to traditional drilling, particularly for those with dental anxiety or deep caries lesions. For adult patients, it offers a less intimidating and more comfortable option, especially for those with sensitive teeth, dental phobias, or complex caries cases. By integrating the babaco-based gel into routine dental care, practitioners can offer a more inclusive and patient-centered approach to caries management, ultimately improving oral health outcomes for patients of all ages. Further research and clinical trials will be essential to fully establish the gel’s long-term efficacy and to promote its widespread adoption in dental practices worldwide [3,39].

#### 2.2.7. Critical Analysis of Potential Limitations

While the study provides valuable insights into the enzymatic activity of babaco latex, it is essential to critically analyze the potential limitations of using a babaco-based gel in clinical practice. One major concern is the variability in enzymatic activity due to differences in fruit maturity, extraction methods, and environmental conditions during cultivation. This variability could lead to inconsistent performance of the gel, making it challenging to standardize for widespread clinical use.

Another limitation is the potential for allergic reactions or adverse effects in patients. Although babaco latex is derived from a natural source, it may still contain compounds that could trigger allergic responses in sensitive individuals. Comprehensive safety studies, including allergenicity testing, are necessary to ensure the gel’s safety for all patient populations.

Moreover, the economic feasibility of producing and distributing the babaco-based gel on a large scale must be considered. The cost of cultivating babaco, extracting the latex, and formulating the gel could be prohibitive, especially if the gel requires specialized storage conditions to maintain its enzymatic activity. Future research should include cost-benefit analyses to determine the gel’s economic viability compared to existing CMCR agents [3].

Finally, the regulatory hurdles for introducing a new dental product must be addressed. The gel would need to undergo rigorous testing and approval processes to meet regulatory standards, which could delay its availability in the market. Collaborative efforts between researchers, industry stakeholders, and regulatory bodies will be essential to navigate these challenges and bring the babaco-based gel to clinical practice.

## 3. Conclusions

The study demonstrated that the babaco-based gel achieved a 78% success rate in caries removal, significantly outperforming the conventional non-rotary mechanical method, which had a success rate of only 14%. Among the extraction methods evaluated, the distillation method yielded the highest efficacy, with a 100% success rate, followed by percolation (85%), Soxhlet (73%), and maceration (53%). These results highlight the potential of babaco-based gels as a minimally invasive alternative to traditional caries removal techniques.

Future research should focus on several key areas to further validate and optimize the use of babaco-based gels. In vivo trials are essential to assess the gel’s performance in a clinical setting, particularly in pediatric patients who may benefit most from minimally invasive approaches. Patient-reported outcomes should be evaluated to understand the gel’s impact on anxiety, pain, and overall patient satisfaction. Microbiological evaluations are necessary to determine the gel’s antimicrobial properties and its effectiveness against cariogenic bacteria.

Additionally, specific tests should be conducted to better understand the gel’s composition and efficacy. These include gas chromatography analysis to identify and quantify bioactive components, proteolytic enzyme quantification experiments to measure enzymatic activity, and animal trials to assess safety and efficacy before proceeding to clinical studies. These steps will provide a comprehensive understanding of the gel’s potential and ensure its clinical and commercial viability.

In conclusion, the babaco-based gel represents a promising advancement in dental caries treatment, offering a less invasive and potentially more patient-friendly alternative to conventional methods. Continued research and development in this area could pave the way for broader clinical applications and improved patient outcomes.

## 4. Materials and Methods

An ex vivo cross-sectional experimental study was carried out in the research and development laboratory of the Catholic University of Cuenca Faculty of Dentistry. The sample comprised 60 teeth, and human teeth were considered inclusion criteria.

### 4.1. Procedure for Obtaining Raw Material

- Raw Material Collection: Fresh babaco (*Carica pentagona*) was harvested directly from the plant provided by the Catholic University of Cuenca, Cuenca, Azuay, Ecuador.
- Sample Processing: The collected babaco was thoroughly washed, dried, and disinfected using antiseptic alcohol. Laminar sections were carefully prepared and placed in pre-sterilized aluminum trays to isolate the second layer of babaco tissue.
- Dehydration: The prepared babaco sections were transferred to an incubator and dehydrated at 60 °C for 48 h.
- Storage: After dehydration, the material was ground into a fine powder using a mortar and stored in airtight plastic tubes at room temperature, ensuring protection from moisture.

### 4.2. Chemical Methods for Obtaining Papain

Extraction techniques for papain involve complex processes that require the use of solvents such as alcohols, hydrocarbons, and chloroalkanes. It is critical to apply these techniques meticulously, using the appropriate solvent and adhering strictly to established protocols to prevent degradation or loss of volatile compounds [40]. The preservation of proteolytic enzymes during extraction can vary significantly depending on the method used, as each technique involves different conditions that may affect enzyme stability, activity, and integrity [41]. According to literature, for preserving proteolytic enzymes, maceration is the most suitable method due to its gentle, room-temperature conditions. Percolation is also a good option if the solvent and extraction time are carefully controlled. Distillation and Soxhlet extraction are generally less favorable due to their reliance on high temperatures, which can denature enzymes. However, specialized low-temperature distillation techniques may be explored for specific applications such as this study [40].

#### 4.2.1. Distillation

- Historical Context: Distillation, a technique pioneered by Egyptian alchemists, employs specialized devices to vaporize and separate volatile substances. Over time, it has become a cornerstone process in the food, cosmetics, and chemical industries due to its ability to purify liquid mixtures.
- Principle: The effectiveness of distillation relies on the differences in boiling points of the components in a mixture. A significant difference in boiling points facilitates the efficient separation of components, yielding a higher degree of purity in the final product [41].

Distillation involves heating a mixture of substances to separate two or more liquids by boiling. This causes the more volatile components to evaporate and then condense in the coolant, returning to a liquid state. As a result, the least volatile components remain in the flask. A variation of this process, known as vacuum distillation, uses rotary evaporators to carry out the separation at temperatures below the boiling point. This is particularly useful for mixtures containing low-volatile solutes, as it reduces the risk of decomposition of the components remaining in the flask. In this method, the most volatile substance evaporates and condenses quickly, while the less volatile substance stays in the flask. However, if both compounds are highly volatile, complete separation cannot be achieved through simple distillation [41,42].

Distillation is a process in which a liquid is heated until its most volatile components transform into vapor. The vapor is then condensed, recovering it in liquid form. The primary objective of distillation is to obtain the most volatile component in its pure form. Process:

- Two clamps are placed on the universal support. The first clamp holds the asbestos mesh on which the pot with water up to half is placed, and the second clamp holds the measuring flask.
- The clamp that will support the coolant is placed on the second universal support, which is joined to the measuring flask using glass tubes and rubber stoppers.
- The lighter is connected to the gas cylinder with the valve and hose. The burner containing the measuring flask is then placed under the pot.
- The reagents are placed in the measuring flask: 200 mL of alcohol at 96° and 20 g of the second layer of crushed babaco. After this, a rubber stopper with a thermometer is placed to control the liquid output.
- The burner is turned on, and the water flow for the coolant is opened.
- A beaker is placed at the end of the coolant to obtain the result of the distillation in a water bath; since it reached a boiling point of 78.3° C, 150 mL of alcohol evaporated, and the distillation ended.
- The solution in the measuring flask is filtered using a funnel and filter paper placed inside it.
- The result of the enzymatic complex is 40 mL and is placed in sterile syringes [42].

It is important to clarify that, in this experiment, a preliminary maceration process was conducted prior to distillation to prevent enzymatic degradation caused by high temperatures. Maceration, which involves soaking the babaco material in a solvent at room temperature, was employed to extract the enzymatic components gently and preserve their activity. This step was crucial because it allowed the subsequent distillation process to be carried out at a lower temperature of 55 °C, minimizing the risk of thermal denaturation of the enzymes. By combining maceration with low-temperature distillation, the enzymatic integrity of the babaco extract was maintained, ensuring the effectiveness of the final gel formulation.

#### 4.2.2. Soxhlet

The Soxhlet extraction method remains one of the most widely used standard techniques for extracting solid samples. In this procedure, the finely ground solid sample is placed in a porous cartridge, which is then positioned in the Soxhlet extractor chamber. The extracting solvent in the flask is heated, producing vapors that condense and drip onto the cartridge containing the sample. This process extracts the soluble analytes from the sample. When the level of condensed solvent in the chamber reaches the top of the lateral siphon, the solvent, now containing the dissolved analytes, flows back into the boiling flask through the siphon. This cycle repeats continuously until the analytes are fully extracted from the sample and concentrated in the solvent [43,44].

Soxhlet extraction offers several advantages: the sample is repeatedly exposed to fresh portions of solvent, and the extraction is performed with hot solvent, which enhances the solubility of the analytes. However, this method also has significant drawbacks, including the lengthy extraction time, the large volume of organic solvent required, and the need for a final solvent evaporation step to concentrate the analytes [44]. Soxhlet process:

- The 20 g sample of babaco is placed on filter paper inside the cartridge, and 200 mL of solvent (96% alcohol) is placed in the flask.
- The Soxhlet apparatus is assembled without forgetting the water inlet and outlet through hoses.
- The heater is turned on, and the process begins. The heater waits for it to complete its automatic cycle but constantly monitors the procedure.
- Once thirty turns have been completed, and the sample is exhausted, the heater is turned off, and the sample is left to rest until it has thoroughly cooled.
- The solvent must be removed to obtain the enzymatic complex, and the distillation method is used.
- The clamp that will support the coolant is placed on the second universal support. This clamp is attached to the measuring flask using glass tubes and rubber stoppers.
- The burner is connected to the gas cylinder using the valve and hose, after which the burner is placed under the pot containing the measuring flask.
- The reagents are placed in the measuring flask: 175 mL of alcohol plus the enzymatic complex resulting from the process. After that, a rubber stopper with a thermometer is placed to control the liquid output.
- The burner is turned on, and the water flow of the coolant is opened.
- Place a beaker at the end of the condenser to obtain the result of the water bath distillation. When the water bath reaches a boiling point of 78.3 °C, the alcohol evaporates, and the distillation ends [44].

#### 4.2.3. Maceration

Maceration is the simplest form of solid-liquid extraction, requiring only a solute, solvent, an amber flask, and a funnel. The process involves adding a volume of solvent to the amber flask that is twice the amount of the solute, ensuring the solvent fully covers the solute. The mixture is then left to rest for 7 days in a dark place at a temperature not exceeding 37 °C. After the resting period, the active ingredient is separated from the solvent by filtering the mixture using a funnel and filter paper [45,46].

The maceration method preserves the natural properties of the solute without altering its composition, making it an effective technique for obtaining extracts in their most authentic form [45]. Maceration process:

- The pulverized babaco is weighed on the laboratory scale, resulting in 34.25 g. This weight is important to determine the amount of soluble needed in the maceration process.
- The crushed raw material is poured into an amber jar, and a double quantity of soluble (96° alcohol) is added to it, for a total of 68.5 mL.
- The amber jar is then covered and wrapped in aluminum foil to prevent light from passing through, and it is left to rest in a cool, dark, and dry place for seven days.
- After the days of rest, the product is filtered using a funnel with filter paper.
- Once the product is filtered, 38 mL of a solution consisting of alcohol and an enzymatic complex rich in *Carica pentagona* is obtained.
- Subsequently, the distillation process is carried out in a water bath to separate the alcohol in the solution obtained.
- Once the distillation process is finished, 15 mL of alcohol and 21 mL of enzyme complex are obtained, which are placed in sterile syringes to preserve them before verifying their effectiveness [45].

#### 4.2.4. Percolation

Percolation implies that the fluid slowly passes through the material’s pores. The process is usually carried out to obtain the soluble part of a solid substance, for which the appropriate solvent must be used [47].

The percolator is a conical container with an upper opening in which a perforated circular lid can be placed. The lid allows the passage of the liquid and subjects the materials placed in it to slight pressure. At the bottom, it has an adjustable closure to allow liquid passage at a convenient speed. Percolation is the least suitable extraction method for highly gelatinous substances or if the drugs are very voluminous [48]. The following steps were taken for the percolation process:

- The percolator equipment is prepared, and a layer of sterile cotton and two paper filters are placed.
- The percentage of active ingredients is then weighed on the scale.
- 25.05 g of the active ingredient and 14.8 mL of 96% alcohol are added until all the raw material is covered.
- Two paper filters are placed to cover the active ingredient, and marbles are placed to apply force.
- It is completely covered with aluminum foil.
- It is left to rest for 3 days.
- On the fourth day, an amber glass is placed on the tip of the bottle, and force is applied to cause the active ingredient to fall.
- The active ingredient is obtained and placed in a completely sterile syringe [48].

### 4.3. Formulation Components

All formulation components were provided by the Catholic University of Cuenca, Cuenca, Azuay, Ecuador.

- 10% hydroalcoholic extract of *Carica pentagona*
- Natrusol: 0.075%
- Methylparaben: 0.05%
- Mint
- Glycerin q.s.p.
- Distilled water q.s.p.

The sample was collected and processed to obtain the hydroalcoholic extract of *Carica pentagona*. One kilogram of immature *Vasconcellea × heilbornii* fruit was collected, washed with potable water, and allowed to dry naturally. The fruits were then carefully selected, separating the pulp from the seeds, and sliced into thin sections. These slices were shade-dried for 7 days, yielding 43.8 g of dehydrated pulp.

The active ingredient was extracted using the maceration method. A mixture of 500 mL of 96% ethyl alcohol and the dehydrated pulp of *Carica pentagona* was prepared and hermetically sealed for 7 days. After this period, the mixture was filtered, and the resulting solution was concentrated using a water bath at a temperature not exceeding 60 °C.

Prior to gel formulation, preliminary tests were conducted to determine the optimal concentration of papain in the hydroalcoholic extract of *Carica pentagona*. Based on existing literature, in vitro studies have evaluated papain concentrations of 2%, 4%, 6%, 8%, and 10% in animal pulp fibroblast cultures. These studies found no statistically significant differences in efficacy across the various concentrations. Consequently, a 10% concentration of the hydroalcoholic extract of *Carica pentagona* was selected for further use.

### 4.4. Preparation and Evaluation of the Papain-Based Gel

A base gel without active components was prepared by boiling 500 mL of distilled water. To this, 7.50 g of Hydroxyethylcellulose (Natrosol™) and 0.05% methylparaben were added (provided by the Catholic University of Cuenca, Cuenca, Azuay, Ecuador), and the mixture was stirred until homogeneous. Subsequently, 25 mL of glycerin and 1 mL of green dye were incorporated, and the mixture was stirred until a uniform gel was achieved. No artificial pH stabilizer was required, as Hydroxyethylcellulose is non-ionic. Once the homogeneous base gel was prepared, the previously obtained hydroalcoholic extract of *Carica pentagona* was added to the formulation.

The final gel formulation underwent a series of tests to ensure it possessed the appropriate physicochemical, rheological, and microbiological properties for its intended use. The key tests included:

- Organoleptic Evaluation: The physical properties of the gel, such as appearance, color, odor, and texture, were assessed. A sample of the gel was examined under adequate lighting against a white background to identify any irregularities. Consistency was evaluated by observing the gel’s flow behavior using a syringe.
- pH Determination: The pH of the final formulation was measured using a pH meter and found to be approximately 7, indicating neutrality.
- Microbiological Tests: Microorganism counting and sterility tests were conducted to confirm the absence of microbiological contaminants and to verify the effectiveness of the preservatives over the product’s shelf life.
- Dental Tissue Adhesion Test: This test evaluated the gel’s ability to adhere to dental tissue for a sufficient duration to exert its therapeutic effect. The test was performed on extracted human teeth, where the gel was applied, and its adhesion time was measured.

### 4.5. Pilot Test

The initial phase of the research aimed to determine whether the latex extracted from the Babaco peel exhibited digestive enzymatic properties. The latex was collected directly by cutting the fruit, and 0.25 mL (five drops) was placed into two test tubes. The following mixtures were prepared:

- Tube 1: 5 drops of milk + 5 drops of latex
- Tube 2: 5 drops of milk + 5 drops of latex + 95% ethanol
- Tube 3: 5 drops of milk + 5 drops of 95% ethanol (negative control)

Results:

Tube 1: The mixture of latex and milk showed protein coagulation, indicating enzymatic activity.

Tube 2: The mixture of latex, milk, and 95% ethanol demonstrated enhanced enzymatic activity.

Tube 3: The mixture of milk and ethanol (negative control) showed no enzymatic activity, confirming that the enzymatic effect was due to the latex.

**Figure 5:**
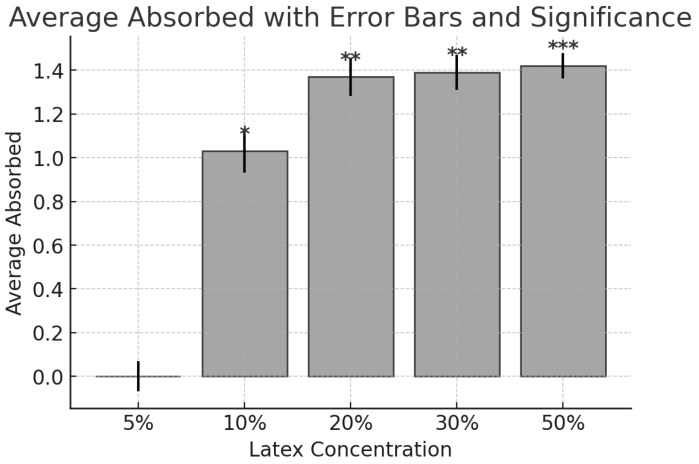
Absorbance levels of babaco latex at varying concentrations. A graph showing the enzymatic activity of babaco latex at concentrations of 5%, 10%, 20%, 30%, and 50%, with 20% identified as the minimum effective concentration. Average Absorbed with Error Bars and Significance. Error bars represent standard deviation (or standard error). Asterisks indicate statistical significance compared to the control group: * (*p* < 0.05), ** (*p* < 0.01), *** (*p* < 0.001).

Based on these findings, various concentrations of latex in ethanol (5%, 10%, 20%, 30%, and 50%) were prepared to determine the minimum concentration required for enzymatic activity. Using a GENESYS™ 20 Spectrophotometer (from Biomedis, provided by the Catholic University of Cuenca, Cuenca, Azuay, Ecuador), the absorbance of each dilution was measured. The results indicated that the enzymatic activity of the 20% and 50% concentrations did not differ significantly. Therefore, 20% was established as the minimum effective concentration for enzymatic activity (Figure 5).

The concentration of the active compound in the final gel formulations was not directly determined. Instead, we relied on previous research and established data to infer the enzymatic activity and concentration of the active compounds in the gel. Acosta’s study found that the highest proteolytic activity was observed in 5-month-old fruits, with an activity of 8125.65 U/g, which decreased as the fruit matured [23,24].

### 4.6. Macroscopic Determination of the Effectiveness in Caries Removal

This gel was applied to half of the cavity of an extracted tooth, and the dentin curettage technique was applied. The other half was also cleaned with curettage but without Babaco gel. Subsequently, observations were conducted using an Olympus SZ61 stereo microscope at 10× magnification (from Biomedis, provided by the Catholic University of Cuenca, Cuenca, Azuay, Ecuador). The analysis revealed a distinct contrast in surface texture between the two treated sides. The side treated with the Babaco gel preparation exhibited a complete absence of roughness, whereas the side treated solely with a curette displayed significant surface irregularities. Based on these preliminary findings, a research initiative was designed in collaboration with undergraduate students to systematically assess the degree of surface roughness using the standardized Sotelo E. scale. This study aims to provide a quantitative evaluation of the observed effects and further elucidate the efficacy of the Babaco gel preparation.

### 4.7. Standardized Gel Application and Experimental Protocol

To ensure the uniform application and consistent performance of the babaco-based gel, a rigorous and standardized protocol was followed. The gel’s consistency was carefully controlled through a series of physicochemical, rheological, and microbiological tests, and its application was meticulously monitored to ensure reproducibility. The gel’s physical properties, including appearance, color, odor, and texture, were assessed under adequate lighting against a white background in an organoleptic evaluation. Any irregularities in consistency were identified and addressed, and the gel’s flow behavior was evaluated using a syringe to confirm it had the appropriate viscosity for dental application. This step ensured the gel could be easily dispensed and would adhere to the dental tissue without dripping or spreading unevenly. The pH of the gel was measured using a pH meter from Biolab provided by the Catholic University of Cuenca, Cuenca, Azuay, Ecuador and found to be approximately 7 (neutral), ensuring compatibility with dental tissues and stability during application, while minimizing the risk of irritation or adverse reactions. Microbiological testing, including microorganism counting and sterility tests, was conducted to confirm the absence of contaminants, ensuring the gel remained stable and effective during application and storage. Additionally, a dental tissue adhesion test was performed on extracted human teeth to evaluate the gel’s ability to adhere to dental tissue for a sufficient duration (60 s) to exert its therapeutic effect, confirming that the gel could maintain contact with the carious tissue without running off or pooling.

The gel was applied using a calibrated syringe to ensure that the exact same amount (0.1 mL) was dispensed into each cavity, maintaining consistency across all samples and ensuring each tooth received an equal dose of the active ingredient. After dispensing, the gel was spread evenly across the cavity surface using a small dental instrument to ensure complete coverage of the carious area, maximizing its effectiveness. The gel was allowed to act for a calibrated and measured time of 60 s before the carious tissue was removed using a dentin spoon excavator. This standardized time frame ensured that the enzymatic action of the gel was consistent across all samples. To reduce bias, the evaluators were blinded to the treatment groups, meaning they were not informed which side of the tooth (left or right) had been treated with the babaco gel or the conventional method. This blinding protocol ensured that the histological and microscopic evaluations were conducted objectively, without preconceived influence from knowledge of the treatment conditions.

The sample size was determined using the Openepi.com 3.01 application, specifically the option to determine the sample size for a cohort study. A 95% bilateral significance level (1-alpha) and 80% power (1-beta) were considered, with 50% in the positive unexposed cell and 95% in the positive exposed cell. The statistical package indicated that 15 samples per group were required according to the Fleiss comparative sample size procedure. The study sample consisted of 60 extracted human teeth, with each tooth divided into two sections. On the right side, the gel was applied using babaco extract obtained by the maceration method (15 teeth), distillation method (15 teeth), Soxhlet extraction method (15 teeth), and percolation (15 teeth). On the left side, the conventional non-rotary mechanical method was used to remove the carious tissue. The gel was applied and allowed to act for 60 s in half of the selected cavity. The carious tissue was then removed using a dentin spoon excavator, with movements made from front to back without going deeper into the cut; only sweeping movements were performed, and the resulting material was removed with gauze. After 30 excavating movements, the treated area was washed with distilled water, and the same number of movements were performed on the other side. Importantly, all procedures were conducted by a single operator and were exactly the same for both sides of each tooth, ensuring consistency and reproducibility throughout the experiment.

Following the completion of the curettage process on both halves, a notable difference in coloration was observed. The side treated with the gel exhibited a color consistent with healthy dentin, whereas the untreated side displayed the characteristic hue of reparative dentin. The specimen was subsequently transferred to the histology laboratory for further processing. It was first embedded in an acrylic mold and subjected to a 10-day rehydration period, followed by decalcification using 5% nitric acid for 96 h. After decalcification, the specimen was embedded in a paraffin block and sectioned using a microtome to achieve slices with a thickness ranging between 4 and 6 μm. These sections were then mounted onto slides and stained with Hematoxylin and Eosin (H&E). A cover slip was applied to prepare the samples for microscopic examination.

The prepared slides were analyzed under an indirect light microscope, with specific focus on the areas treated with the Babaco gel and those treated solely with the dentin spoon. The regions treated with Babaco gel demonstrated uniform and consistent removal of carious tissue, reflecting a smooth and regular surface. In contrast, the areas treated only with the dentin spoon exhibited irregular surfaces, with uneven removal of carious tissue and minor superficial marks resembling the effect of a shovel scraping wet soil. These findings suggest that the Babaco gel facilitates a more controlled and precise removal of caries compared to the conventional method.

### 4.8. Histological Evaluation of Chemomechanical Removal

The specimens were subsequently transferred to the oral pathology laboratory at the Universidad Peruana Cayetano Heredia, where they were systematically categorized and coded based on the extraction method used for the active ingredient. The specimens were divided into four distinct groups, each assigned a unique identifier for organizational and analytical purposes:

Transparent Box Group: This group comprised specimens in which the active ingredient was extracted using the Soxhlet method. The study units within this group were labeled numerically, ranging from 001 to 016.

Pink Box Group: Specimens in this group underwent extraction of the active ingredient via distillation. Each sample was coded alphabetically, using letters such as A, B, C, D, and so on.

Blue Box Group: In this group, the active ingredient was extracted through maceration. The teeth in this category were coded with the names of countries, such as Afghanistan, Brazil, and others.

Orange Box Group: This group included specimens where the active ingredient was extracted using the percolation method. Each sample was coded with the names of colors, such as yellow, blue, white, etc.

This systematic coding and categorization ensured clear identification and facilitated efficient analysis of the specimens based on their respective extraction methods. To eliminate potential observer bias, the pathologist conducting the analysis was blinded to the treatment assignments. Specifically, the pathologist was not informed which side of the specimen had been treated with the gel and which side had undergone the alternative treatment. This blinding protocol ensured that the evaluation of the specimens was conducted objectively, without any preconceived influence from knowledge of the treatment conditions. Such measures are critical to maintaining the integrity and reliability of the experimental results.

## Figures and Tables

**Table 1:** Comparison of caries removal success rates with and without babaco gel application. A table summarizing the success rates of caries removal for teeth treated with babaco gel versus those treated with the conventional method, categorized by extraction method (maceration, percolation, distillation, and Soxhlet).

Success in Caries Removal					
Gel Formulation			Without Gel Formulation		
	N	Success Rate (%)	N	Success Rate (%)	Sig.
MACERATION *n* = 15	8	53%	5	33%	0.3008 *
PERCOLATION *n* = 13	11	85%	3	23%	0.013 **
DISTILLATION *n* = 15	15	100%	0	0%	N.A.
SOXHLET *n* = 15	11	73%	0	0%	N.A.
TOTAL *n* = 58	45	78%	8	14%	<0.01 *
			Chi-square *		Fisher **

* = Chi-square test was used. ** = Fisher’s exact test was used; result is significant. N.A. = No statistical test was performed due to lack of variability in the data.

**Table 2:** Post Hoc comparison of caries removal success rates by extraction method. A table presenting the results of a Post Hoc test comparing the success and failure rates of caries removal across the four extraction methods (distillation, maceration, percolation, and Soxhlet), with statistical significance (*p* = 0.019).

Post Hoc Comparison						
			Gel Formulation		Total	Post Hoc Group
			Success	Failure		
Extraction Method	Distillation	Count	15	0	15	A
		Expected	11.6	3.4	15.0	
		Residual	2.4	−2.4		
	Maceration	Count	8	7	15	C
		Expected	11.6	3.4	15.0	
		Residual	−2.6	2.6		
	Percolation	Count	11	2	13	B
		Expected	10.1	2.9	13.0	
		Residual	0.7	−0.7		
	Soxhlet	Count	11	4	15	B
		Expected	11.6	3.4	15.0	
		Residual	−0.5	0.5		
Total		Count	45	13	58	
		Expected	45.0	13.0	58.0	

Chi square *p* = 0.019.

## Data Availability

All data and materials are available or request from the corresponding author. The data are not publicly available due to ongoing research using a part of the data.

## Abbreviations

The following abbreviations are used in this manuscript:

ART	Atraumatic Restorative Treatment
CMCR	Chemomechanical Caries Removal
